# Effects of cognitive training under hypoxia on cognitive proficiency and neuroplasticity in remitted patients with mood disorders and healthy individuals: ALTIBRAIN study protocol for a randomized controlled trial

**DOI:** 10.1186/s13063-024-08463-5

**Published:** 2024-10-03

**Authors:** Kamilla Woznica Miskowiak, Viktoria Damgaard, Johanna Mariegaard Schandorff, Julian Macoveanu, Gitte Moos Knudsen, Annette Johansen, Pontus Plaven-Sigray, Claus Svarer, Caroline Bruun Fussing, Katrine Cramer, Martin Balslev Jørgensen, Lars Vedel Kessing, Hannelore Ehrenreich

**Affiliations:** 1 NEAD Centre, Psychiatric Centre Copenhagen, Frederiksberg Hospital, Psychiatric Centre Copenhagen, Mental Health Services, Hovedvejen 17, Frederiksberg, Capital Region of Denmark DK-2000 Denmark; 2 Department of Psychology, University of Copenhagen, Øster Farimagsgade 2A, Copenhagen, DK-1353 Denmark; 3 Neurobiology Research Unit, Copenhagen University Hospital, Rigshospitalet, Copenhagen, Denmark; 4 Copenhagen Affective Disorder Research Centre (CADIC), Psychiatric Centre Copenhagen, Psychiatric Centre Copenhagen, Mental Health Services, Frederiksberg, Denmark; 5 Department of Clinical Medicine, University of Copenhagen, Copenhagen, Denmark; 6 Clinical Neuroscience, Max-Planck-Institute of Experimental Medicine, City Campus, Göttingen, Germany; 7 Department of Psychiatry and Psychotherapy, Central Institute of Mental Health, Medical Faculty Mannheim, Heidelberg University, Mannheim, Germany

**Keywords:** Hypoxia, Cognition, Neuroplasticity, Cognitive remediation, Depression, Bipolar disorder

## Abstract

**Background:**

Cognitive impairment is prevalent across neuropsychiatric disorders but there is a lack of treatment strategies with robust, enduring effects. Emerging evidence indicates that altitude-like hypoxia cognition training may induce long-lasting neuroplasticity and improve cognition. We will investigate whether repeated cognition training under normobaric hypoxia can improve cognitive functions in healthy individuals and patients with affective disorders and the neurobiological underpinnings of such effects.

**Methods:**

In sub-study 1, 120 healthy participants are randomized to one of four treatment arms in a double-blind manner, allowing for examination of separate and combined effects of three-week repeated moderate hypoxia and cognitive training, respectively. In sub-study 2, 60 remitted patients with major depressive disorder or bipolar disorder are randomized to hypoxia with cognition training or treatment as usual. Assessments of cognition, psychosocial functioning, and quality of life are performed at baseline, end-of-treatment, and at 1-month follow-up. Functional magnetic resonance imaging (fMRI) scans are conducted at baseline and 1-month follow-up, and [^11^C]UCB-J positron emission tomography (PET) scans are performed at end-of-treatment to quantify the synaptic vesicle glycoprotein 2A (SV2A). The primary outcome is a cognitive composite score of attention, verbal memory, and executive functions. Statistical power of ≥ 80% is reached to detect a clinically relevant between-group difference with minimum *n* = 26 per treatment arm. Behavioral data are analyzed with an intention-to-treat approach using mixed models. fMRI data is analyzed with the FMRIB Software Library, while PET data is quantified using the simplified reference tissue model (SRTM) with centrum semiovale as reference region.

**Discussion:**

The results will provide novel insights into whether repeated hypoxia cognition training increases cognition and brain plasticity, which can aid future treatment development strategies.

**Trial registration:**

ClinicalTrials.gov, NCT06121206. Registered on 31 October 2023.

**Supplementary Information:**

The online version contains supplementary material available at 10.1186/s13063-024-08463-5.

## Background

Cognitive impairment across memory, attention, and executive functions is a core dimension of several neuropsychiatric disorders that persists after remission, hampers recovery, and contributes to socio-occupational disability [1–3], which is the largest socio-economic burden of the disorders [4]. This highlights cognitive impairment as a pressing treatment target to improve the lives of patients and reduce societal costs. However, there are no approved treatments with efficacy on cognition in affective disorders, including bipolar disorder (BD) and major depressive disorder (MDD) [5, 6], or in schizophrenia [7]. Cognitive remediation interventions produce overall small to moderate cognitive benefits according to recent meta-analyses in affective disorders [8] and schizophrenia [9], but a large proportion of patients show no improvement [10]. A breakthrough in treatment strategies seems only possible if we move beyond the current understanding of brain processes involved in cognitive health and disease.

Neuroplasticity is the ability of the brain to adjust to challenges by forming new synapses, dendritic spines, and connections and modifying circuit wiring. It is of fundamental importance for cognitive functions and its aberration is involved in cognitive impairments across neuropsychiatric disorders [11]. Many studies have investigated pharmacological treatments acting on pathways involved in neuroplasticity but show only preliminary results [5–7, 11]. Recently, “plasticity-based” computerized cognitive training was found to increase markers of neuroplasticity [12] and cognition in schizophrenia [13], albeit with mixed results [13, 14]. A key challenge that impedes discovery of cognition treatments with robust and long-lasting efficacy is the incomplete understanding of mechanisms underlying *enduring* neuroplasticity.

The fundamental importance of oxygen for animal life has been understood for centuries. William G. Kaelin Jr., Sir Peter J. Ratcliffe, and Gregg L. Semenza obtained in 2019 the Nobel Prize for their discovery of hypoxia-inducible factors (HIF), key transcription factors that regulate gene expression in response to decreases in cellular oxygen availability. Among the HIF regulated genes is erythropoietin (EPO), encoding a potent (hematopoietic) growth factor and, consequently, leading to increased red blood cell production. However, little is known about how oxygen manipulation influences brain plasticity and cognitive functions. In contrast with the lay view that low ambient oxygen has only adverse effects on the central nervous system, *compiling evidence indicates otherwise*. Exposure of neural progenitor cells to low oxygen increases their numbers, survival, and maturation [15]. Imitating hypoxia by stabilizing HIF also improves cognition in healthy mice [16]. Intermittent *moderate* reduction in ambient oxygen (O_2_) from 21% (sea-level) to 9–16% (corresponding to the partial pressure at ≈ 2100–6700 m altitude) enhances the recovery of limb function and respiration after spinal cord injury in animals and humans, effects that are augmented by task-specific training [17, 18]. In a recent systematic review, we examined the evidence for benefits of moderate hypoxia interventions on cognition, neurological functioning, and markers of neuroplasticity across 8 human and 48 animal studies [19]. A key finding was that repeated exposure to lower doses of moderate hypoxia (10–16% O_2_) for 30–240 min over several weeks, ideally in conjunction with physical exercise or motor-cognitive training, yielded neuroprotective effects and potential cognitive benefits. However, larger and methodologically robust translational studies are needed to further validate these findings [19, 20].

Erythropoietin (EPO) is a protein that, in addition to its effects on red blood cell production, plays a fundamental, evolutionarily ancient role in neurodevelopment, neuroprotection, and cognition. EPO and its receptor (EPOR) are present also in insects where they facilitate neuroprotection [21, 22]. In mammals, EPO and EPOR are expressed in hippocampus and cortex and are upregulated upon brain injury or hypoxia [23], which is an endogenous neuroprotective mechanism [23]. Systemically injected EPO crosses the intact blood-brain barrier also in humans and has neuroprotective and neurotrophic effects [23–25]. We found some evidence that EPO can improve cognition in healthy participants and patients with MDD, BD [26–33], schizophrenia, or multiple sclerosis [34, 35], and that this was accompanied by reversal of hippocampal volume loss [28, 31, 33] and enhanced task-related frontoparietal activity [30, 32]. Nevertheless, a major challenge is the cost of EPO and lack of support for clinical trials by EPO producing companies. Thus, alternative ways to increase brain EPO may instead represent a therapeutic breakthrough. We recently discovered that three-week constant moderate hypoxia (12%) combined with motor-cognitive training increases learning capacity and neuroplasticity in mice by inducing mild neuronal hypoxia and upregulation of brain EPO and EPOR [36]. We suggest that this is a key mechanism of long-lasting neuroplasticity and cognitive improvement. While this is an important lead, the discovery of therapeutic effects in animal models provides insufficient prediction of efficacy in humans [11]. A crucial next step is thus to test the efficacy in multidisciplinary human intervention studies.

## Aims and hypotheses

In the ALTIBRAIN project, we will conduct two parallel randomized controlled studies in patients with affective disorders who exhibit cognitive impairments and in healthy participants. Specifically, we will investigate whether three weeks repeated altitude-like hypoxia cognition training (low ambient oxygen and cognitive training) leads to increase in cognitive performance, brain function, and neuroplasticity, effects similar to those seen with intravenous EPO treatment [28, 31, 33–35, 37]. Based on our preclinical studies [36, 38], we expect a long-lasting cognitive improvement accompanied by increase in hippocampal volume and function and in presynaptic density in the hippocampus and prefrontal cortex. Specifically, the ALTIBRAIN trial will test the following hypotheses in healthy participants and in patients with MDD or BD: (i) altitude-like hypoxia cognition training will induce robust cognitive improvement (primary outcome); (ii) this effect will be accompanied by increases in presynaptic density and in regional brain volume and activity measured with positron emission tomography (PET) SV2A [^11^C]UCB-J binding and magnetic resonance imaging (MRI), respectively; (iii) hypoxia with no training and normoxia with training will each induce small transient cognitive increase.

## Methods

### Study design and procedure

**Fig. 1:**
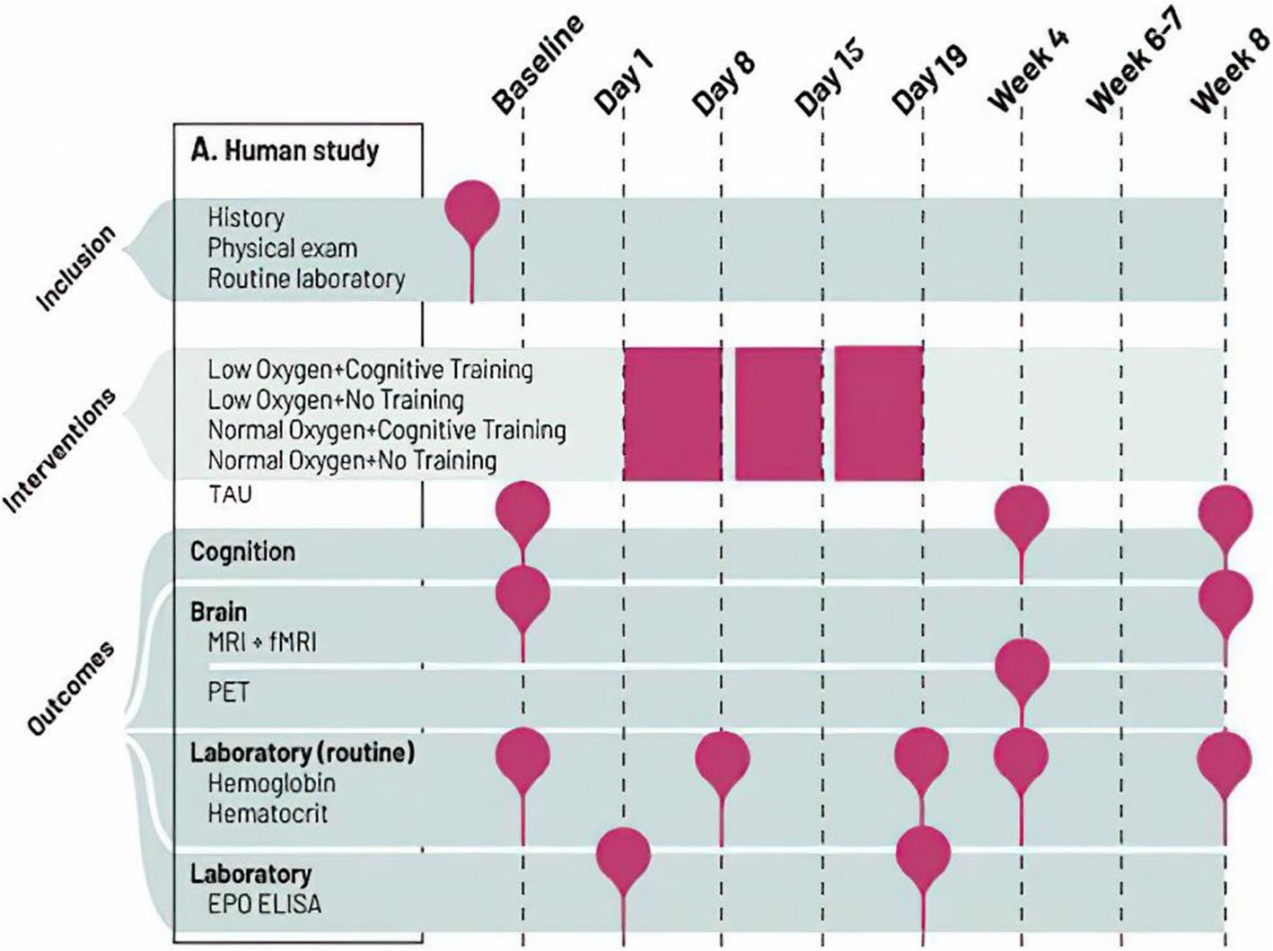
Overview of events across parallel sub-studies

In a basic scientific study in healthy humans, we will determine the causal mechanisms of changes in neuroplasticity and cognition across multiple levels of analysis (sub-study 1). The clinical study (sub-study 2) will in parallel clarify whether targeting these mechanisms translates into cognitive benefits in cognitively impaired patients, in line with a phase II study design. In this way, both studies will provide proof-of-concept evidence for potential benefits of intermittent hypoxia combined with cognitive training on brain plasticity and cognitive functioning*.* Figure 1 depicts an overview of study events.

Sub-study 1 involves four intervention groups: (1) normobaric hypoxia (12% O_2_) combined with cognitive training, (2) hypoxia (12% O_2_) with no training, (3) cognitive training under normoxia (20% O_2_), and (4) normoxia (20%) with no training in a double-blinded controlled design. This design enables us to disentangle combined and separate effects of altitude-like hypoxia and cognitive training. Participants are randomized in blocks and undergo interventions in these groups for practical reasons. During the three-week treatment, participants breathe 12% ambient oxygen (≈ 4400 m altitude) or approximately normal sea-level oxygen (20%) in a treatment room, 3.5 h daily, six days per week (18 sessions in total). After a blood test (days 1, 6, 20) and light standardized breakfast, they enter the treatment room. Here, they sit at a desk separated by desk dividers. On iPads, they perform cognitive training or matched control games without cognitive benefits. Cognitive training is interleaved by short breaks, during which the participants can relax or walk on a treadmill inside the room. This is to avoid complete physical inactivity throughout the 3.5 h sessions and thereby make the sessions more pleasant for participants. Participants undergo cognition assessments in weeks 1 (baseline), 4, and 8 and functional and structural MRI in weeks 1 and 8, when red blood cell counts are comparable between groups. Participants in the two extreme groups (normobaric hypoxia with cognitive training and normoxia with no training) will also undergo PET scanning in week 4 (post-treatment). Participants will be recruited consecutively for the PET scans until an equal distribution in each of the extreme intervention arms (i.e., hypoxia with cognitive training and normoxia with no training), sex, and age group is achieved.

Sub-study 2 involves two intervention groups: (1) normobaric hypoxia (12%) combined with cognitive training 3.5 h daily, five to six days per week for three weeks (16 sessions in total), and (2) treatment as usual (TAU) in an assessor-blind controlled design. Patients in the TAU group will initially be assessed at the same time points as the active group with neurocognitive testing at baseline, week 4, and week 8, fMRI at baseline and week 8, and PET in week 4. After completed testing at week 8, patients in the TAU group undergo the three-week active intervention, followed by one additional neurocognitive assessment and mood rating in the week after treatment completion. The inclusion of this TAU arm rather than an active control group was chosen to ensure patient motivation and minimize attrition in this time-intensive study. Similar study designs with TAU control groups are common in pro-cognitive intervention studies investigating novel treatments in mood disorders and schizophrenia [39, 40]. Although patients will not be blinded to their allocated treatment arm, neurocognitive test results are generally not susceptible to placebo effects [41, 42] and assessments will be carried out by blinded, fully trained research assistants (psychology students).

### Participants

For sub-study 1, we will recruit 120 healthy participants with no current or prior psychiatric illness. For sub-study 2, we will recruit 60 patients with affective disorders (MDD or BD) in partial or full remission (Hamilton Depression Rating Scale 17-items (HDRS-17; [43]) and the Young Mania Rating Scale (YMRS; [44]) score ≤ 14). Healthy participants are primarily recruited across the University of Copenhagen and other higher education facilities in Copenhagen to ensure that participants are able to take time off for the time-intensive study. Patients are mainly recruited through the Mental Health Services, Capital Region of Denmark, and through advertisements on relevant websites.

For sub-study 1, eligible participants are between 18 and 50 years of age, be fluent in Danish, and have no psychiatric history. For sub-study 2, patients are between age 18 and 65, be fluent in Danish, have a diagnosis of BD or MDD confirmed using the Schedules for Clinical Assessment in Neuropsychiatry (SCAN) [45], in partial or full remission (defined as a score of ≤ 14 on HDRS-17 and YMRS with objectively verified cognitive impairment according to Screen for Cognitive Impairment in Psychiatry (SCIP) [46] and/or self-reported cognitive impairment measured with Cognitive Complaints in Bipolar disorder Rating Assessment (COBRA) [47]. For SCIP, their performance must be > 0.5 standard deviations (SD) below their demographically adjusted expected total SCIP score or on minimum two SCIP subtest scores [48]. For COBRA, patients must report substantial cognitive impairment defined as a score ≥ 14 [47, 49]. We include a subjective measure of cognitive impairment as eligible patients can have suffered a cognitive decline from a high level of premorbid functioning, which may not be detectable by a cross-sectional evaluation with SCIP.

Common exclusion criteria for both sub-studies are schizophrenia or schizoaffective disorder, organic mental disorders (ICD-10 codes F00-09), history of neurological disorder (including dementia), alcohol or substance abuse, daily use of ≥ 22.5 mg oxazepam, or history of serious head trauma. To ensure safety, candidates are also excluded if they have previous altitude sickness, significant medical conditions (e.g., heart disease, diabetes, renal failure, untreated/insufficiently treated hypertension, and/or thrombosis), history of epilepsy or thromboembolic events, first-degree family with thromboembolic events before age 60, are pregnant or breastfeeding, currently take iron supplements, smoke, use other nicotine products regularly, or have a BMI > 30. All female participants who are not using hormonal contraceptives must take a pregnancy test before beginning the intervention to ensure that they are not pregnant. Participants are also excluded if they have received electroconvulsive therapy (ECT) three months prior to participation or are dyslexic. Regarding fMRI assessments, participants are not eligible if they suffer from claustrophobia and have a pacemaker and/or other metal implants inside their bodies. Participants are not eligible for PET assessments if they have participated in experiments with radioactivity (> 10 mSv) within the last year, have significant occupational exposure to radioactivity, or if they take medication incompatible with study aims (e.g., SV2A binding agents). Patients with mood disorders, who do not meet the fMRI and PET inclusion criteria, will not be excluded from the trial per se, but only from these neuroimaging assessments. All participants must provide written informed consent. The SPIRIT reporting guidelines were used for the current study [50]. See Appendix A for a completed SPIRIT checklist.

### Study setting

The ALTIBRAIN trial, including all neurocognitive assessments and intervention sessions, will be conducted at the NEAD (Neurocognition and Emotion in Affective Disorders) Centre, Department of Psychology, University of Copenhagen, and Psychiatric Center Copenhagen, Frederiksberg Hospital, Denmark. Furthermore, MR and PET scans are conducted at the Copenhagen University Hospital, Rigshospitalet, Copenhagen, Denmark.

### Randomization and blinding

Randomization is conducted in the database program REDCap [51], which is also used to store the collected data, utilizing a 1:1 allocation ratio. Randomization occurs after inclusion of the participants planned to undergo treatment sessions together. Treatment groups are stratified for age (> 27 in sub-study 1 of generally young volunteers/ > 35 in sub-study 2 of patients). For randomization purposes, date of birth is registered to determine the stratum to which the participant belongs at the time of enrolment. Study identification numbers are given consecutively within each stratum and treatment arm. Randomization is conducted in the randomization module in REDCap, where an un-blinded researcher inputs the stratum and the program then retrieves the allocated intervention. To ensure blinding of the outcome-assessors, the randomization module in REDCap is only accessible to the researchers, who are not involved in evaluation of the efficacy parameters.

Healthy participants in sub-study 1 are blind to the oxygen condition since the low/normal oxygen air blown into the room is of equal temperature and humidity. Furthermore, they are told that they receive one of two forms of cognitive training, which effectively blinded participants in a previous study [52]. After study completion, we will assess whether blinding has been maintained for each participant, since there is a small risk that some participants develop symptoms of mild altitude sickness which could unblind them to their intervention. Blinding of the outcome assessors is ensured by their absence during the training visits (where oxygen levels are displayed and monitored on a screen outside the room). Participants will be instructed not to disclose any information concerning their intervention during outcome assessments and under no circumstances will the allocation be revealed to the outcome assessors. The training visits are conducted by researchers who are not involved in outcome assessments.

### Intervention: hypoxia cognition training

#### Low/normal ambient oxygen

**Fig. 2:**
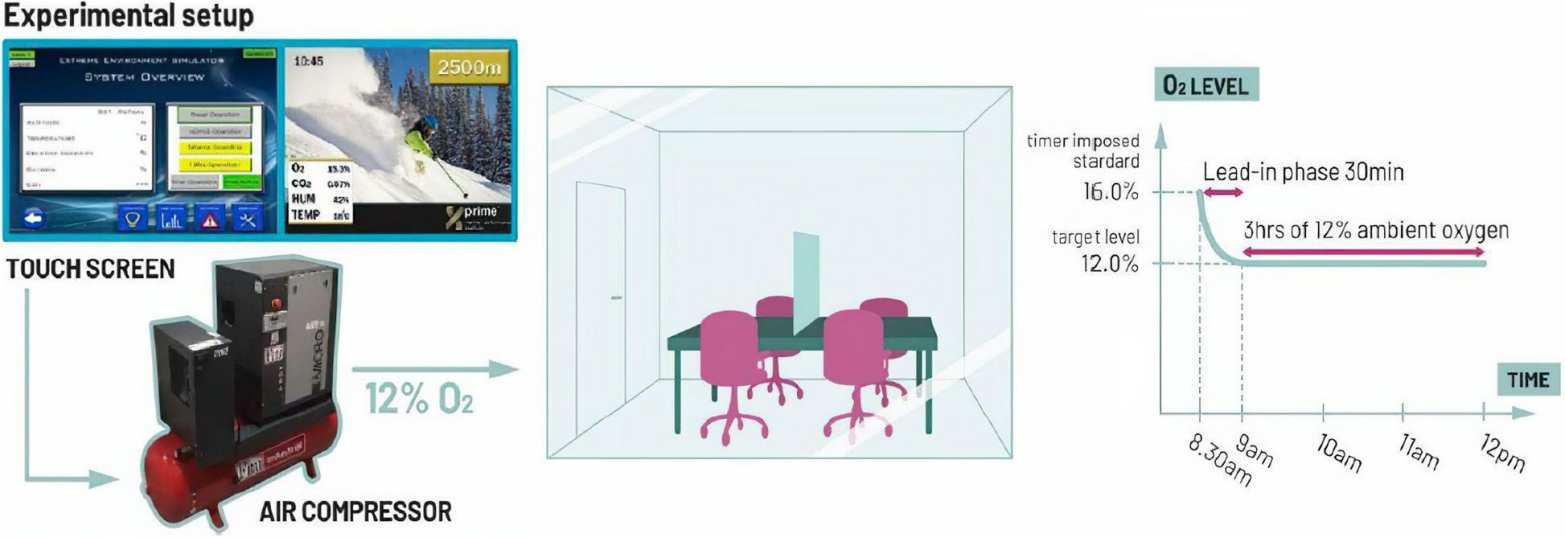
Experimental setup

Fresh air with 12% or 20% O_2_ is blown into a sealed 20 m^3^ room by a 4 kW air compressor with a safety-approved system developed by HöhenBalance, Austria (see Fig. 2 for experimental setup). For the active condition, participants enter the room at 16% O_2_ (≈ 2,200 m altitude). Upon entry, the O_2_ levels will be reduced from 16 to 12% (≈ 4400 m altitude) in a 30-min lead-in phase. The precise control by the HöhenBalance systems allows control of O_2_ levels to ± 0.1%. The target O_2_ level of 12% will be maintained over three hours.

HöhenBalance systems create simulated altitude by introducing normobaric breathable hypoxic air. Therefore, even under any conceivable worst-case failure condition, the systems cannot give rise to any potentially harmful oxygen condition to persons inside or outside the room. A password access control provides additional safety.

#### Cognitive training and matched computer games

The web-based cognitive training (Happy Neuron Pro) is grounded on principles of neuroplasticity-based learning by being intensive, neuroadaptive, engaging, and rewarding. This web-based training program has been translated into Danish and is used for traumatic brain injury, schizophrenia, and in our previous cognitive remediation trial ([53] and www.happyneuronpro.com), making its implementation feasible. Participants in the no training control condition receive computer games similar to Happy Neuron Pro but with low cognitive demand that produce no cognitive benefits [52]. Specifically, this sham procedure involves the exact same stimuli as the active condition but with changes from trial to trial only in the appearance of the tasks. In contrast, the active training involves parametric task adjustment by decreasing stimuli presentation time, increasing working memory load, decreasing time to respond, and increasing the number of non-target items (distractors).

### Outcome measures

For an overview of outcome assessment domains, measures, frequency, and timing, see Table 1. The outcome measures listed below are consistent with the latest recommendations from the International Society for Bipolar Disorders (ISBD) Task Force [54].

**Table 1:** Schedule of enrolment, interventions, and assessments

**Timepoint**	**Pre-intervention**	**Baseline (week 0)**	**Active treatment phase**	**End of treatment (week 4)**	**Follow-up (week 8)**	**Additional end of treatment assessment (week 12)** For sub-study 2 TAU group
**Enrolment**						
Project information: written/oral communication	x					
Written informed consent	x					
Eligibility assessment	x					
Randomization	x					
**Intervention and safety outcomes**						
Repeated moderate hypoxia exposure or normoxia (3.5 h per day, five to six days per week for three weeks)			x			
Cognitive training or sham training (3.5 h per day, five to six days per week for three weeks)			x			
Blood sampling (blood counts)	x	x	x (days 1, 8, and 19)	x	x	
Safety monitoring and assessments of potential adverse events		x	x	x	x	/
Mood ratings (HDRS + YMRS)	x	x		x	x	/
ESQ			x			
VAS			x			
**Cognitive measures**						
RAVLT		x		x	x	/
TMT-A		x		x	x	/
TMT-B		x		x	x	/
RBANS Coding		x		x	x	/
RBANS Digit Span		x		x	x	/
WAIS-III LNS		x		x	x	/
RVP		x		x	x	/
SWM		x		x	x	/
OTS		x		x	x	/
ERT		X		x	x	/
Verbal fluency (S + D)		x		x	x	/
SDMT		x		x	x	/
WCST		x		x	x	/
**Functioning measures**						
FAST		x			/	
CAVIR		x			x	/
**Self-report measures**						
AQoL		x		x	x	/
COBRA		x		x	x	/
SDS		x		x	x	/
WHOQOL-BREF		x		x	x	/
WSAS		x		x	x	/
PSQI		x		x	x	/
**Neuroimaging**						
sMRI + fMRI		x			x	
PET (a subgroup of the participants)				x		
**Other measures**						
DART		x				
CTQ	x					
SCIP	x					
Research biomarker blood samples			x (days 1 and 19)			

x = included in both sub-studies. / = included in sub-study 2 only

*Abbreviations*: *HDRS* Hamilton Depression Rating Scale, *YMRS* Young Mania Rating Scale, *ESQ* Environmental Symptoms Questionnaire, *VAS* visual analogue scale, *RAVLT* Rey Auditory Verbal Learning Test, *TMT-A* Trail Making Test Part A, *TMT-B* Trail Making Test Part B, *RBANS* Repeatable Battery for the Assessment of Neuropsychological Status, *WAIS-III LNS* Wechsler Adult Intelligence Scale version 3 Letter-number-sequencing, *RVP* Rapid Visual Information Processing (CANTAB, Cambridge Cognition Ltd.), *SWM* spatial working memory (CANTAB, Cambridge Cognition Ltd.), *OTS* One Touch Stockings of Cambridge (CANTAB, Cambridge Cognition Ltd.), *ERT* Emotion Recognition Task (CANTAB, Cambridge Cognition Ltd.), *SDMT* Symbol Digit Modalities Test, *WCST* Wisconsin Card Sorting Test, *Connor’s CPT* Connor’s Continuous Performance Test, *FAST* Functional Assessment Short Test, *CAVIR* Cognition Assessment in Virtual Reality test, *AQoL* Assessment of Quality of Life, *COBRA* Cognitive Complaints in Bipolar disorder Rating Assessment, *SDS* Sheehan Disability Scale, *WHOQOL-BREF* World Health Organization Quality Of Life, *WSAS* Work and Social Adjustment Scale, *PSQI* Pittsburgh Sleep Quality Index, *fMRI* functional magnetic resonance imaging, *PET* positron emission tomography, *DART* Danish Adult Reading Test, *CTQ* Childhood Trauma Questionnaire, *SCIP* Screen for Cognitive Impairment in Psychiatry (Danish version)

#### Primary outcome measure

The primary outcome measure is changes in a cognitive composite score, consisting of neuropsychological tests covering attention, memory, and executive functions from baseline to week 4. We have previously demonstrated an improvement on this *speed of complex cognitive processing* composite measure in patients with BD after 8 weeks of EPO treatment [28]. In the present trial, the specific tests included in the primary composite outcome measure are the Rey Auditory Verbal Learning Test (RAVLT) [55], The Repeatable Battery for the Assessment of Neuropsychological Status (RBANS) Coding [56], Verbal Fluency with the letter “D” [57], Wechsler Adult Intelligence Scale (WAIS)-III Letter-Number Sequencing [58], Trail Making Test Part B (TMT-B) [59], and Rapid Visual Information Processing (RVP) from the Cambridge Neuropsychological Test Automated Battery (CANTAB, Cambridge Cognition Ltd.). To derive the cognitive composite score, we will z-transform and sum performance scores from RAVLT total recall, TMT-B, WAIS-III Letter-Number Sequencing, RBANS Coding, Verbal Fluency (letter “D”), and RVP speed for correct responses [28].

#### Secondary outcome measures

The secondary cognitive outcome measure consists of mean choices to correct in the One Touch Stockings of Cambridge (OTS) test from CANTAB, which yielded particularly strong effects of cognitive training with Happy Neuron in our previous study [60]. Dorsal prefrontal cortex (dPFC) activity during spatial working memory (N-back task) is another secondary outcome measure because the dPFC is consistently engaged in both healthy participants and patients with mood disorders in response to successful pro-cognitive interventions [61].

Additionally, sub-study 2 of patients with mood disorder includes a measure of daily functioning as secondary outcome in accordance with the ISBD recommendations [54]. The effect of the intervention on daily functioning is assessed with a novel virtual reality test of daily life cognitive skills, the Cognition Assessment in Virtual Reality (CAVIR) test [62]. The CAVIR test is an engaging, immersive, and self-administered 360° VR test in a kitchen, where the participant’s cognitive skills related to planning and preparing a meal are assessed, thus enabling insight into patients’ daily life cognitive skills.

#### Tertiary outcome measures

The tertiary cognitive outcome measures are the RAVLT, RBANS Coding and Digit Span, Verbal Fluency with the letters “S” and “D”, WAIS-III Letter-Number Sequencing, the Wisconsin Card Sorting Task (WCST), the Rapid Visual Information Processing (RVP; CANTAB), additional measures from the One Touch Stockings of Cambridge (OTS; CANTAB), the spatial working memory (SWM; CANTAB), and the Emotion Recognition Test (ERT; CANTAB), as well as the Trail Making Test Part A (TMT-A) and B (TMT-B), respectively.

The Assessment of Quality of Life (AQoL) [63], the Cognitive Complaints in Bipolar Disorder Rating Assessment (COBRA) [47], Sheehan Disability Scale (SDS) [64], the World Health Organization Quality of Life (WHOQOL-BREF) [65], the Work and Social Adjustment Scale (WSAS) [66], and the Pittsburgh Sleep Quality Index (PSQI) [67] will be applied. Finally, psychosocial functioning is also assessed with the clinician-rated interview Functional Assessment Short Test (FAST) [68]. History of early life stress will be assessed with the Childhood Abuse and Trauma Scale [69] at the time of inclusion.

To minimize learning effects on neuropsychological test with repeated testing at the follow-up assessments, alternate versions of the RAVLT (original list AB, GeAB, and Cr-AB) and RBANS Coding and Digit Span (version A and B) are used. These versions are administered in counter-balanced order within each stratum. Finally, premorbid verbal IQ is assessed with the Danish Adult Reading Test [70].

#### Additional mechanistic outcome measures

Cognition-related neural activity will be assessed with resting state fMRI and the following carefully selected fMRI tasks: (i) a spatial N-back working memory task and (ii) a novel grocery list strategic encoding paradigm. Alternate matched versions of the grocery list fMRI paradigm are used at the two testing times to minimize neural desensitization to the test stimuli. Structural MRI measures of interest are hippocampal and cortical volume, cortical thickness, and white matter integrity. The PET measure of interest is hippocampal and prefrontal cortex SV2A [^11^C]UCB-J binding as a readout of presynaptic density, as stated in the aims.

To further increase insight into the underlying neurobiological mechanisms involved in the potential beneficial cognitive effects of hypoxia cognition training, blood samples from first day (day 1) and second to last day of treatment (day 19) will be analyzed for the exploratory purpose of investigating whether baseline levels and/or changes of peripheral biomarkers, including EPO, are correlated with cognitive improvement.

Blood based biomarkers of neuroplasticity (e.g., brain derived neurotrophic factor), inflammation (e.g., interleukin factors), and EPO will be obtained at baseline (BL) and end of treatment for explorative analyses.

#### Biochemistry

Blood test data is pseudo-anonymized by the Clinical Biochemistry Department—Section for External Projects, Copenhagen University Hospital. This is done by producing serial numbers that are attached to the individual blood tests and a separate electronic conversion key linking the serial numbers to participants ID numbers. Research blood samples will be transferred to the Neuropsychiatric Laboratory, Frederiksberg Hospital, and stored at − 80 °C until use. The collected blood samples will be stored until they are analyzed in a research biobank set up for this project. Measurements will be performed at Department of Clinical Pharmacology, Rigshospitalet. The research biobank will cease at the end of the project (expected date 01 January 2029), and any excess material will then be transferred to Cimbi Biobanken, which is an existing and approved biobank for future research under the Neurobiological Research Unit, Copenhagen University Hospital, Rigshospitalet.

### Statistical analyses

#### Primary, secondary, tertiary, and exploratory outcome measure analyses

Behavioral data from neuropsychological test score performance, subjective cognitive impairments, quality of life, level of functioning, psychosocial functioning, and mood symptoms (i.e., data from the primary, secondary, and tertiary outcomes) will be analyzed using mixed model design. Hence, the analyses investigate how the mean score of each outcome changes over time in each treatment group and testes for interaction effects (i.e., are the trajectories different between the groups). All analyses will be based on an intention-to-treat (ITT) principle. Hence, all participants will be analyzed based on their allocated intervention arm. Participants with incomplete data will also be included in the analyses without any ad hoc imputation [71]. No interim analyses will be performed. A nonblinded researcher will pseudo-anonymize the data prior to analysis, and data analyses will thus be conducted by blinded researchers.

#### MRI analyses

Whole-brain and hippocampal volume and cortical thickness will be accessed using the FMRIB Software Library (FSL) vertex-based analysis and FreeSurfer Image Analysis Suite, respectively. White matter integrity is assessed with tract-based spatial statistics.

Resting state and task-based functional MRI (fMRI) data will be preprocessed and analyzed with the FMRIB Expert Analysis Tool (FEAT; latest version available at trial completion) part of FMRIB’s Software Library (FSL; www.fmrib.ox.ac.uk/fsl). For resting-state fMRI data, we will use Multivariate Exploratory Linear Optimized Decomposition into Independent Components (MELODIC) to conduct group-level ICA by multi-session temporal concatenation, identifying the common resting state networks across the groups. Furthermore, fMRI data from the N-back working memory task and the grocery list encoding and retrieval tasks will be analyzed using a region-of-interest analysis to assess differences between the intervention and control groups in neural activity in the dorsal PFC (dPFC) after completing 4 weeks of treatment (adjusting for any difference in neural activity at baseline). We will also conduct volume-of-interest analyses of the dPFC for the N-back task and for both the dPFC and hippocampi for the word encoding task to investigate our hypotheses. Finally, exploratory whole-brain analyses will be conducted to investigate any treatment-related effects in other brain regions.

#### Positron emission tomography analyses

Dynamic [^11^C]UCBJ-J PET imaging will be acquired over 90 min using the High Resolution Research Tomograph (HRRT; CTI/Siemens, Knoxville, TX, USA) PET scanner as previously described [72]. Following motion-correction, the PET data are processed using the PVElab software pipeline (https://nru.dk/index.php/allcategories/category/30-software), or similar, where PET images are co-registered to participants’ T1-weighted 3 T MR image, and regions of interest are automatically delineated [73].

Radioactivity concentrations are extracted from grey matter to generate regional time-activity curves (TACs) and subsequently used for kinetic modeling. Quantification is performed using the simplified reference tissue model 2 (SRTM2) with the white matter region centrum semiovale as pseudo-reference tissue. The outcome is the non-displaceable binding potential (BP_ND_; ratio of specifically bound radioligand to that of non-displaceable radioligand in tissue).

### Sample size and power calculation

Power calculations were performed with the software program G*Power 3.1.9.4 [74] and were based on the primary hypothesis that altitude-like hypoxia combined with cognitive training produces robust sustained cognitive improvement compared with normoxia and no training (hypothesis i). As hypoxia triggers the endogenous production of EPO, we based the power calculation on our previous clinical study of subcutaneous EPO treatment in which we demonstrated clinically relevant cognitive enhancement [75]. In that study, the differential change between EPO and placebo groups in the same cognitive composite score as we apply in the present study was 0.5 standard deviations (SD) [75]. Assuming an effect of 0.4 SD differential change (medium effect size) between the two groups and 0.5 SD of the change, we will achieve > 80% power to detect an effect at an alpha level of 0.05 by including 26 participants per group. This number of participants provides adequate power for the structural and functional MRI investigations based on our previous studies of EPO [30, 32, 33]. To accommodate for up to a 20% drop-out, we will include 30 participants per treatment arm; i.e., 120 participants in sub-study 1 and 60 patients in sub-study 2.

For PET imaging of SV2A [^11^C]UCB-J binding (reflecting presynaptic density), power calculations involved the following assumptions: (i) the average (SD) *BP*_ND_ in the frontal cortex in healthy participants is 3.36 (0.38) (based on in-house data), and (ii) the combined intervention will lead to a 10% increase in *BP*_ND_. With these assumptions, we will achieve a power of 0.82 to detect an effect at significance level of 0.05 post-treatment of the combined intervention (primary aim) with *n* = 20 per treatment arm using a two-tailed, independent-sample *t*-test.

### Data management and monitoring

All personal information will be obtained at the eligibility assessment or from patient records in cases where participants are unable to provide the needed information. Pseudo-anonymized data from the neuropsychological tests, virtual reality test, questionnaires, interviews, and functional assessment will be registered in the REDCap database, which meets requirements from the Danish Data Protection Agency. Pseudo-anonymized raw paper data from neuropsychological tests are kept in a locked filing cabinet. Questionnaires and interviews are collected directly in REDCap. Data quality is ensured by score range restrictions on values for all outcomes, and all data for the primary and secondary outcome measures will be doublechecked prior to analyses. Signed consent forms as well as a list that matches participant ID numbers with personal information are kept separate from pseudo-anonymized data. The key matching participants’ personal information with their ID number will be deleted and consent forms maculated 10 years after study completion. At this point, all data will be completely anonymized. All trial authors will have access to the final trial dataset. If a participant is excluded or withdraws from the study, the reason for exclusion is documented in REDCap, along with information regarding any adverse events.

### Participant retention

In accordance with the Danish National Scientific Ethics Committee’s guidelines for remuneration or other benefits to voluntary participants, all healthy participants (sub-study 1) will receive remuneration corresponding to their time participating in neuropsychological examinations, neuroimaging, and treatment sessions. Patients (sub-study 2) will be offered feedback on the results of their neuropsychological assessments after completing the final assessment. In accordance with the Danish National Scientific Ethics Committee’s guidelines for remuneration or other benefits for patient participants, they will receive gift cards for neuropsychological examinations and neuroimaging. In addition, all participants in both studies will receive transport allowance in connection with the various study days.

## Discussion

Cognitive impairment across domains is highly prevalent in several neuropsychiatric disorders, which impedes recovery and psychosocial functioning. A key challenge that hampers discovery of cognition treatments with robust and long-lasting efficacy is the incomplete insight into the mechanisms underlying long-lasting neuroplasticity. Emerging evidence suggests cognitive benefits of moderate hypoxia training in humans and animal studies [76], possibly through increased EPO expression triggered by hypoxia [36]. Nevertheless, there are no well-controlled studies with multiple outcomes that also includes mechanistic translational work. The objective of the present trial is therefore to investigate the effects of a three-week hypoxia (12% O_2_) cognition training intervention in healthy individuals (sub-study 1) and remitted patients with affective disorders (sub-study 2) on cognition, daily functioning, and brain function and neuroplasticity. The results will provide insights into potential pro-cognitive effects of hypoxia cognition training and may influence future treatment strategies targeting cognition and neuroplasticity across brain disorders.

### Strengths

The present trial involves a multidisciplinary research approach that capitalizes on the relative strengths of each approach. In basic scientific studies in healthy humans (sub-study 1), we will elucidate the causal mechanisms of changes in neuroplasticity and cognition across multiple levels of scientific analysis from presynaptic density measured with PET and neurocircuitry network activity measured with fMRI to neurocognitive tests. Furthermore, the clinical study (sub-study 2) will clarify whether targeting these mechanisms translates into cognitive and functional benefits in cognitively impaired patients with mood disorders. With the four treatment arms in the basic scientific study (sub-study 1), we will be able to determine the combined—potential synergistic—effects of hypoxia and cognitive training and the separate effects of these treatment modalities. The trial also complies with the expert recommendations by the ISBD Cognition Task Force to select a broad cognitive composite score spanning attention, memory, and executive function as *primary outcome* in cognition trials in mood disorders [54]. Furthermore, the inclusion of self-reported, observer-rated, and performance-based measures of functioning will provide insight into whether potential cognitive benefits of EPO translate into improved daily functioning, which is the ultimate goal for our patients. Finally, the use of neuroimaging and blood-based measures will provide mechanistic insights into potential cognitive improvement induced by hypoxia cognition training, in line with international recommendations [61].

### Limitations

The extensive somatic illness exclusion criteria limits generalizability of findings. However, these exclusion criteria are necessary to ensure participant safety of this novel repeated hypoxia training intervention, which is of principal importance. In particular, a large proportion of patients with mood disorders have somatic comorbidities. Therefore, if the present trial reveals positive effects on cognition, important next steps would be to examine the safety of hypoxia training in patients with somatic comorbidities. Furthermore, the included patients with BD and MDD are mostly on psychotropic medication. While this supports generalizability of the findings, since we want to target residual cognitive impairments in symptomatically stable patients who are mostly medicated, these concomitant medications may also confound [54] cognitive test, PET, and fMRI results [77]. To minimize confounding effects, patients’ medication will be kept stable during the study participation period, if possible, and by accounting for medication status in post hoc analyses. In addition, since the healthy group is primarily recruited from University of Copenhagen, it is possible that they have less capacity than patients for cognitive and synaptic changes. Finally, it is a potential limitation that we based our power calculations on the effect size demonstrated in a study of EPO treatment rather than a hypoxia study, which may be more directly comparable. However, the expected change of 0.4 SD in cognition corresponds to a clinically relevant cognitive enhancement as defined by the ISBD task force [7].

### Study feasibility

While our recent mouse studies [36, 38] provide a firm basis for ALTIBRAIN, and our longstanding work on EPO treatment of cognitive impairment across animals and humans lends significant support, the translation of findings from animals to humans is often poor [11]. There is therefore a risk that we find no substantial cognitive improvements in humans. In this case, our multidisciplinary approach Notwithstanding the risk, we evaluate the chances of detecting treatment-related cognitive improvements in humans as high based on our previous findings that pro-cognitive effects of EPO treatment seemed to translate from rodents to humans [25–28, 30–33, 78–80] and were accompanied by brain changes as assessed with functional and structural MRI across healthy human participants [26, 27] and patients with neuropsychiatric disorders [24, 28, 31, 34] and that similar functional neuronal changes were observed with intensive cognitive training [81].

### Safety procedures and monitoring of hypoxia training

The procedure involves repeated exposure to normobaric 12% O_2_, which corresponds to an altitude of 4400 m. Similar daily repeated exposures to simulated altitudes of 4–8000 m over days to weeks are used widely, including by the US army, to induce high-altitude acclimatization and minimize risk of altitude sickness upon subsequent ascend to high altitudes [82]. We therefore evaluate the risk of altitude sickness in our study as minimal. Nevertheless, as safety precautions, we will (i) apply a 30-min lead-in phase, where oxygen levels are reduced slowly from 16 to 12% to allow for physiological adjustment; (ii) exclude individuals with risk factors for altitude sickness; (iii) monitor potential symptoms of altitude sickness at a daily basis with the Environmental Symptoms Questionnaire (ESQ) related to cerebral function [83]; and (iv) collaborate with medical doctors at the Frederiksberg Hospital (LVK, MBJ, CBF) as in our previous EPO intervention studies. We will report the mean ESQ scores in the primary outcome articles in addition to any adverse events that may occur during the intervention.

### Ethical considerations

A key ethical consideration is the extensive time requirement of participants over a period of three weeks. Given this, we will recruit primarily university students in the first study, since they are likely to have time. For patients with affective disorders, the extensive time requirement may be overwhelming. Therefore, we decided to give patients up to two days off (Saturday and Sunday) every week, (in contrast with only one day off for healthy participants, leading to a total of 16—as opposed to 18 in sub-study 1—treatment sessions over three weeks. We are monitoring patients’ mood state at continuous basis with daily brief monitoring of subjective state and any adverse reactions through the VAS and ESQ questionnaires and weekly mood ratings assessed with HDRS and YMRS, respectively. If patients show mood symptom exacerbation to a score of ≥ 18 on the HDRS or YMRS, they are followed up with one more rating after two days, and if no improvement is seen, they are referred to their psychiatrist and drop-out is considered. In our experience, patients with affective disorders generally find it meaningful to take part in cognition trials because of their intrinsic motivation to try new treatments that may reduce their debilitating cognitive difficulties. This has so far ensured high adherence to and satisfaction with study participation.

To minimize attrition and ensure motivation, we have chosen an online cognitive training program and matched computer games that are highly engaging, rewarding, and—for the active condition—neuroadaptive (for more details, see [52]). Finally, fMRI is non-invasive, and PET [^11^C]UCBJ will be associated with small exposure to radioactivity, not much more than the annual background radiation. PET [^11^C]UCBJ has been found safe in studies examining synaptic density in healthy adults [84], in adolescents [71] and in patients with Alzheimer’s disease [85] or depression [86].

To ensure participant safety, all personnel on the project are certified in assessing and addressing suicidal thoughts and behavior through their completion of a suicide prevention course provided by the Capital Region of Denmark (must be renewed at a regular basis). If suicidal thoughts or behavior are detected during a mood rating, the clinician will perform a thorough screening for suicidal risk and contact the participant’s doctor. In acute situations, the clinician will escort the suicidal patient to a psychiatric emergency room via taxi. It is thereby ensured that any participant with mood symptoms, including suicidal thoughts and behavior during their participation in the study is cared for responsibly. Every participant is further under the coverage of the public insurance institution, the Patient Compensation Association.

### Perspectives

This is a project that has the potential to unravel a novel mechanism of enduring neuroplasticity and long-term improvement in cognitive functions, which can contribute to the current understanding of brain plasticity and unlock novel effective treatments for cognitive decline. With the multidisciplinary approach, the project can lead to a mechanistically informed therapeutic approach targeting cognitive impairments across patients with neuropsychiatric disorders.

Version and date identifier: Protocol article version 1, 22 April 2024.

## Trial status and dissemination

Recruitment commenced in February 2023 and is planned to be completed in June 2025. Sixty-two participants in sub-study 1 and 33 participants in sub-study 2 were included in the trial as of April 2024. The project will result in 5-6 articles published in peer-reviewed international scientific journals. The planned publications will involve all the scientific collaborators who contributed significantly to the study with the PhD students (VD and JM) receiving first authorships on the primary outcome paper of sub-studies 1 and 2, respectively. The core scientific team (VD, JM, KWM, LVK, HE) will have co-authorships on all subsequent papers including potential post-hoc analyses and will include the PI (KWM) as senior author. All results will be published whether negative, inconclusive, or confirming the project hypotheses. Author eligibility will be assessed using the Vancouver Convention, and there will be no utilization of professional writers. Protocol article version 1, 22 April 2024.

## Supplementary Information

Additional file 1: Appendix A. SPIRIT 2013 Checklist: Recommended items to address in a clinical trial protocol and related documents.

## Data Availability

The datasets used and analyzed in the current study are available from the corresponding author upon reasonable request.

## Abbreviations

ABCR: Action-Based Cognitive Remediation
AQoL: The Assessment of Quality of Life
BD: Bipolar disorder
BMI: Body mass index
CANTAB: Cambridge Neuropsychological Test Automated Battery
CAVIR: Cognition Assessment in Virtual Reality
COBRA: Cognitive Complaints in Bipolar Disorder Rating Scale
DART: Danish Adult Reading Test
DMN: Default mode network
dPFC: Dorsal prefrontal cortex
ECT: Electroconvulsive therapy
EPO: Erythropoietin
ERT: Emotion recognition test
ESQ: Environmental Symptoms Questionnaire
FAST: Functional Assessment Short Test
FEAT: FMRIB Expert Analysis Tool
fMRI: Functional magnetic resonance imaging
FSL: FMRIB Software Library
HIF: Hypoxia inducible factor
HDRS-17: Hamilton Depression Rating Scale – 17 items
ISBD: International Society for Bipolar Disorder
ITT: Intention-to-treat
MRI: Magnetic resonance imaging
NEAD: Neurocognition and Emotion in Affective Disorders Centre
O_2_: Oxygen
OTS: One Touch Stockings of Cambridge
PET: Positron emission tomography
RAVLT: Rey Auditory Verbal Learning Test
RBANS: Repeatable Battery for the Assessment of Neuropsychological Status
RVP: Rapid Visual Information Processing
SCAN: Schedules for Clinical Assessment in Neuropsychiatry
SCIP-D: Screen for Cognitive Impairment in Psychiatry – Danish version
SD: Standard deviation
SDS: Sheehan Disability Scale
SV2A: Synaptic vesicle glycoprotein 2A
SWM: Spatial working memory
TAU: Treatment as usual
TMT-A: Trail Making Test A
TMT-B: Trail Making Test B
UD: Unipolar disorder
VR: Virtual reality
WAIS-III LNS: Wechsler Adult Intelligence Scale Version III Letter–Number Sequencing
WCST: Wisconsin Card Sorting Task
WHOQOL-BREF: World Health Organization’s Quality of Life Assessment
WSAS: Work and Social Adjustment Scale
YMRS: Young Mania Rating Scale
